# Morpho-chemical characterization of individual ancient starches retrieved on ground stone tools from Palaeolithic sites in the Pontic steppe

**DOI:** 10.1038/s41598-023-46970-8

**Published:** 2023-12-07

**Authors:** G. Birarda, E. Badetti, C. Cagnato, G. Sorrentino, I. Pantyukhina, C. Stani, S. Dal Zilio, G. Khlopachev, S. Covalenco, T. Obada, N. Skakun, A. Sinitsyn, V. Terekhina, A. Marcomini, C. Lubritto, N. Cefarin, L. Vaccari, L. Longo

**Affiliations:** 1 Elettra-Sincrotrone Trieste, S.S. 14 - km 163,5 in Area Science Park, 34149 Basovizza, Trieste Italy; 2 Department of Environmental Sciences, Informatics and Statistics, Ca’ Foscari University of Venice, Via Torino 155, 30172 Mestre, VE Italy; 3 UMR 8096 Archéologie des Amériques, CNRS, Université Paris 1 - Panthéon-Sorbonne, Paris, France; 4 UMR7268 Anthropologie Bio-Culturelle, Droit, Ethique et Santé (ADES), Marseille, France; 5 Department of Physics, University of Turin, Via Pietro Giuria 1, 10125 Turin, Italy; 6 Institute of History, Archaeology and Ethnology, Far-Eastern Branch, IHAE-FEB RAS, Vladivostok, Russia; 7 CERIC-ERIC, S.S. 14 - km 163,5 in Area Science Park, 34149 Basovizza, Trieste Italy; 8 CNR IOM, S.S. 14 - km 163,5 in Area Science Park, 34149 Basovizza, Trieste Italy; 9 Peter the Great Museum of Anthropology and Ethnography (the Kunstkamera) of the Russian Academy of Science, St. Petersburg, Russia; 10 Institute of Cultural Heritage, Academy of Sciences of Moldova, Chişinău, Moldova; 11 Institute of Zoology, National Museum of Ethnography and Natural History of Moldova, Chişinău, Moldova; 12 Institute for the History of Material Culture, IHC-RAS, St. Petersburg, Russia; 13 Department of Environmental, Biological and Pharmaceutical Sciences and Technologies, University of Caserta “Luigi Vanvitelli”, Via Vivaldi 43, 81100 Caserta, Italy; 14 ADM School, Nanyang Technological University, Singapore, Singapore

**Keywords:** Infrared spectroscopy, Conservation biology, Cheminformatics, Anthropology, Archaeology

## Abstract

Despite the extensive literature on the retrieval of digestible starches from archaeological contexts, there are still significant concerns regarding their genuine origin and durability. Here, we propose a multi-analytical strategy to identify the authenticity of ancient starches retrieved from macrolithic tools excavated at Upper Paleolithic sites in the Pontic steppe. This strategy integrates the morphological discrimination of starches through optical microscopy and scanning electron microscopy with single starch chemo-profiling using Fourier transform infrared imaging and microscopy. We obtained evidence of aging and biomineralization in the use-related starches from Palaeolithic sites, providing a methodology to establish their ancient origin, assess their preservation status, and attempt their identification. The pivotal application of this multidisciplinar approach demonstrates that the macrolithic tools, from which starches were dislodged, were used for food-processing across the Pontic Steppe around 40,000 years ago during the earliest colonization of Eurasia by *Homo sapiens*.

## Introduction

The consumption of dietary carbohydrates has been documented since the Middle Pleistocene by isolating starch grains from dental calculi and coprolites^1^, both considered as providing direct evidence of hominin diets. In contrast, mechanical processing (e.g., pounding, grinding, pestling, etc.) of starch-rich plants before consumption are not well-documented across the Late Pleistocene, although these activities may leave behind use-related biogenic residues^2^ (U-RBRs) on the active surfaces of the pebbles used as ground stone tools (GSTs). Proving the intentional processing of carbohydrate-rich plants through the analysis of potential GSTs is still controversial and often limited by the object biography and possible contamination that may be due to modern starches occurring during activities carried out in the field (excavations), throughout conservation procedures in museum settings, and even in laboratory settings^3,4^.

U-RBRs are comprised by two main types of residues: amorphous, lacking recognizable structure^5^, and structured residues such as pollen, phytoliths, fibers, and starch grains that can be identified through imaging. Among these structured biogenic residues, our focus is on identifying ancient starch grains dislodged from the surfaces of utilized ground stones dating back to 40 ka. We examined samples that came from macrolithic ground stones tools (GSTs) retrieved in three sites across the Pontic Steppe attributed to the early phases of the Upper Paleolithic: Brînzeni I, a cave facing the Prut River basin in Moldova, Surein I, a rock shelter on the southern slopes of Crimea, and from layer III of Kostenki 14-Markina Gora, an open air site along the Don River^6^. These sites represent three different environmental conditions which might have influenced the conservation of the structured U-RBRs (SU-RBRs).

Starch grains exhibit a wide range of dimensions, from a few microns to a hundred microns in width, and various shapes, with the most common ones being round, lenticular, and polygonal. They are composed of approximately 98–99% of two types of α-glucans, amylose and amylopectin, with their ratio varying depending on the plant species. These characteristics reflect their botanical origin and serve as valuable diagnostic indicators, especially for economically relevant taxa that have already been extensively studied^7,8^. Morphological identification of starch grains, both modern and ancient, is conventionally conducted using optical microscopy (OM) at magnifications up to 800X, employing both bright field (unpolarized) and polarized light. Scanning Electron Microscopy (SEM) greatly enhances the detailed observation of starch grain morphology and provides better insights into their preservation status from a structural perspective^7,9,10^. However, sole morphological identification is not sufficient to confirm their genuine ancient origin and remove doubts regarding the possibility of modern contamination^11^.

In this context, various attempts have been made to chemically identify amorphous residues on flaked stone tools using vibrational spectroscopies, such as Raman spectroscopy^12^ and Fourier Transform InfraRed (FTIR) spectroscopy^13–15^, to demonstrate the presence of fatty acids and/or proteins. However, similar approaches have not yet been applied to identify structured U-RBRs, such as starch grains that adhere to the utilized surfaces of GSTs. Furthermore, the preservation of the chemical identity of ancient starches is also unlikely. Prolonged contact with soil constituents can lead to various biodegradation pathways, suggesting that ancient starches may possess a ‘microfossil’ nature that has yet to be revealed. A study involving Fourier-Transform Ion Cyclotron Resonance Mass Spectrometry (FTICR-MS) was conducted on modern starches to analyze the products of their non-enzymatic degradation through the Maillard reaction. This study demonstrated the potential to characterize starchy residues and their diagenetic products, and to recognize taxonomic signals, opening up new possibilities for investigating fossil contexts^16^. Nevertheless, the aforementioned analysis was conducted only on extracts of modern starches and not on ancient ones, which are usually much less abundant, severely limiting the application of the method.

Based on the above, the innovative multi-technique approach proposed in this study aims to address this significant research gap by integrating conventional OM with SEM, Fourier Transform Infrared imaging and microscopy (FTIRI, FTIRM) to enable the identification of SU-RBRs at the individual starch grain level. Distinctive chemical features that discriminate genuine ancient starches from potential modern contaminants have been identified, primarily associated with biomineralization events. This demonstrates the long-term resistance of the structured residues and suggests possible aging pathways^16–18^. Additionally, a chemometric model was developed based on starches extracted from modern plants available in the Pontic steppe, as identified through the literary review and available pollen lists for the sites (see Supplementary materials). This model was utilized for the preliminary classification of the ancient starch grains, with a majority identified as belonging to rhizomes and tubers. Overall, the methodology described in this study addresses two key aspects. First, it addresses issues regarding starch degradation and aging due to diagenetic events and their long-lasting survival. Furthermore, it demonstrates the genuine origin of starch grains obtained from GSTs dating back to at least 36,000 years ago, offering potential for their classification. From an archaeological perspective, these results are likely to demonstrate the co-occurrence of intentional processing of starchy plants with the emergence of soft technologies connected to the use of non-flaked/macrolithic tools since the early occurrence of *Homo sapiens* at the boreal latitudes. This contributes to our understanding of the complexity of modern human dietary habits, as their skills enabled the mechanical transformation of bio-based polymer composites, including starch, and in turn likely strengthening their capacity to overcome the constraints as a result of the harsh climatic conditions of the late MIS3 (Marine Isotope Stage).

## Results

The characterization of the limited number of starches isolated from GSTs requires for non-destructive morpho-chemical analytical approaches with single-starch sensitivity. To this aim, here we propose the exploitation of FTIR, both imaging and infrared synchrotron radiation (IRSR) microscopy, and its integration with OM, both unpolarized and polarized, and SEM for the analysis of putative ancient starches recovered from the used surfaces of nine ground stones retrieved at three sites across the Pontic Steppe: Brînzeni I (Moldova), Surein I (Crimea), and Kostenki 14-Markina Gora (Don River, Russia). More details on the geographical context of the research and the archaeological sites, in addition to the object biographies, are reported in the Supplementary Materials. Table 1 summarizes identifier, excavation site, origin and intended use of the examined GSTs. Supplementary Fig. 1 shows OM images of selected GSTs. A sketch of the flowchart describing the cleaning of the GSTs and U-RBRs extraction is presented in Supplementary Fig. 5.

**Table 1:** Investigated GSTs: archaeological site, site type, tool identifier and synthetic description-intended use. The sampled GSTs are stored in museum collections.

Archaeological site	Site type	Tool identifier	Synthetic description^4,6,19^	GST attributed function
Surein I (Crimea)	Rockshelter	#NN†	Large oval GST, square 9B, lowermost layer III, limestone	Grinding stone (passive tool)
Brînzeni I (Moldova)	Cave	# 442	Subrectangular fragment of a large GST, fragment that refits with BZ#NN, square 12 g, lowermost layer III, quartz-arenite	Grinding stone (passive tool)
Brînzeni I (Moldova)	Cave	# NN†	Fragment of a large GST, (refits with BZ#442), square 11j, lowermost layer III, quartz-arenite	Grinding stone (passive tool)
Brînzeni I (Moldova)	Cave	# 833	Large fragment that refits with BZ#2965, square 12 g, lowermost layer III, quartzite	Pestle (active tool)
Brînzeni I (Moldova)	Cave	#2965	Small fragment refits with BZ#833, square 12 g, lowermost layer III, quartz-arenite	Pestle (active tool)
Brînzeni I (Moldova)	Cave	#3539	Small oval GST, square 16 e, lowermost layer III, greywacke	Grinding stone (passive tool)
Brînzeni I (Moldova)	Cave	#6707	Large rectangular GST, lowermost layer III, quartz-arenite	Grinding stone (passive tool)
Brînzeni I (Moldova)	Cave	#177	Large broken GST, square 9 i, lowermost layer III, quartz-arenite	Grinding stone (passive tool)
Kostenki 14, Markina-Gora (Russia)	Open air site	#35	Small GST, layer III, L33 214–224, quartzite	Grinder/pestle (active tool)

^†^When the accession number is not available, the specimens are listed as # NN, No Number.

From here on, the analyzed SU-RBRs will be referred to as Ancient Starch Candidates (ASC), and ranked from ASC1 to ASC4 according to the dislodgement procedures detailed elsewhere^4,6,19^. The origin of ASCs, a brief description of their isolation procedure and the analytical techniques exploited for their characterization are summarized in Table 2. The same information is reported in Table 2 also for Modern Starch References (MSRs), extracted in water from underground and above storage organs (USOs and ASOs, respectively) of wild plants selected among those potentially available during the study conditions, according to the literary review of the relevant pollen lists^4,6,19–21^ and also ethnobotanical sources^22^, with the aim to build a chemometric model for ASCs classification.

**Table 2:** Acronym and short description (Origin, Dislodgment procedures, Brief description and applied characterization techniques) of the investigated ancient SU-RBR (i.e., ASC-1 to ASC-4) and MSR samples.

Acronym	Origin	Dislodgment procedures^4,6,19^	Brief description	Characterization techniques used
ASC-1	Brînzeni I, Surein I, Kostenki 14	Molding with Provil L (polyvinyl siloxane) creates the peel-off effect of the adhering residues and a copy of the stone surface to detect wear-traces	Starch grains were observed only by means of SEM, scanning directly the molds peeled off from the used areas of the GST	SEM
ASC-2	Brînzeni I	The thin encrustations adhering onto the GST used surfaces were powdered and sonicated in ultrapure water	The suspensions were deposited on ZnSe windows and analyzed^19^	SEM, FTIRI
ASC-3	Brînzeni I, Surein I, Kostenki 14	Material aliquots were isolated by direct sonication of part of the molds (by indentation) in ultrapure water	The suspensions were deposited on ZnSe or Si windows and analyzed^6,19^	SEM, FTIRI
ASC-4	Brînzeni I, Surein I, Kostenki 14	Selected surfaces of the GST were sonicated in ultrapure water; the suspensions were stabilized with a few drops of EtOH and the solution was processed according to published protocols^23^ in two different labs (Paris and Vladivostok). The procedure allows for the extraction of isolated starch grains	The suspensions were deposited on a glass slide to be analyzed by OM^6,19^. In a second instance, the starches were recovered from the glass slide by suctioning in 50 μL of DI water and dropped onto CaF_2_ or ZnSe windows for SEM and IRSR-FTIR analysis^6,19^	OM, SEM, IRSR-FTIRM
MSR	Pre-Alps (Northern Italy)	Starch grains extracted in water from modern plants available in the Pontic steppe, selected according to literary review	The suspensions were deposited on a glass slide to be analyzed by OM and on ZnSe windows for SEM and FTIRM analysis^6,19^	OM, SEM, FTIRM

Meaning of acronyms: OM, Optical Microscopy; SEM, Scanning Electron Microscopy; FTIRI, Fourier Transform Infrared Imaging; FTIRM, Fourier Transform Infrared Microscopy with conventional IR glow-bar source; IRSR FTIRM, Fourier Transform Infrared microscopy with infrared synchrotron radiation.

Next, the application of the very same characterization techniques is first presented on MSR with the aim of guiding the reader on the more complex interpretation of morpho-spectroscopic results on ASCs.

### Morpho-chemical characterization of modern starches by OM, SEM and FTIRM

As specified in Table 3, six species of USO and eight species of ASO were analyzed by OM, SEM and FTIRM. Among the USOs, two non-Eurasian taxa were included in the dataset, as a suitable reference for common modern contamination agents^24^.

**Table 3:** USO and ASO MSR list: Scientific name and part of the plant tested.

	ASO			USO	
	Scientific name	Part of the plant		Scientific name	Part of the plant
1	*Aesculus hippocastanum*	Seed	1	*Cyperus esculentus*	Rhizome
2	*Brassica oleracea*	Stem	2	*Erythronium dens-canis*	Rhizome
3	*Carex* sp.	Seed	3	*Pueraria lobata*	Root
4	*Gingko biloba*	Kernel	4	*Sagittaria sagittifolia*	Rhizome
5	*Juniperus* sp.	Fruit	5	*Typha latifolia*	Rhizome
6	*Linum usitatissimum*	Seed			
7	*Panicum miliaceum*	Seed			
8	*Quercus ilex*	Kernel			
9	*Trapa natans*	Seed			

	USO				
	Scientific Name of potential contaminants	Part of the plant			
1	*Ipomoea batatas*	Tuber			
2	*Manihot esculenta*	Tuber			

**Figure 1:**
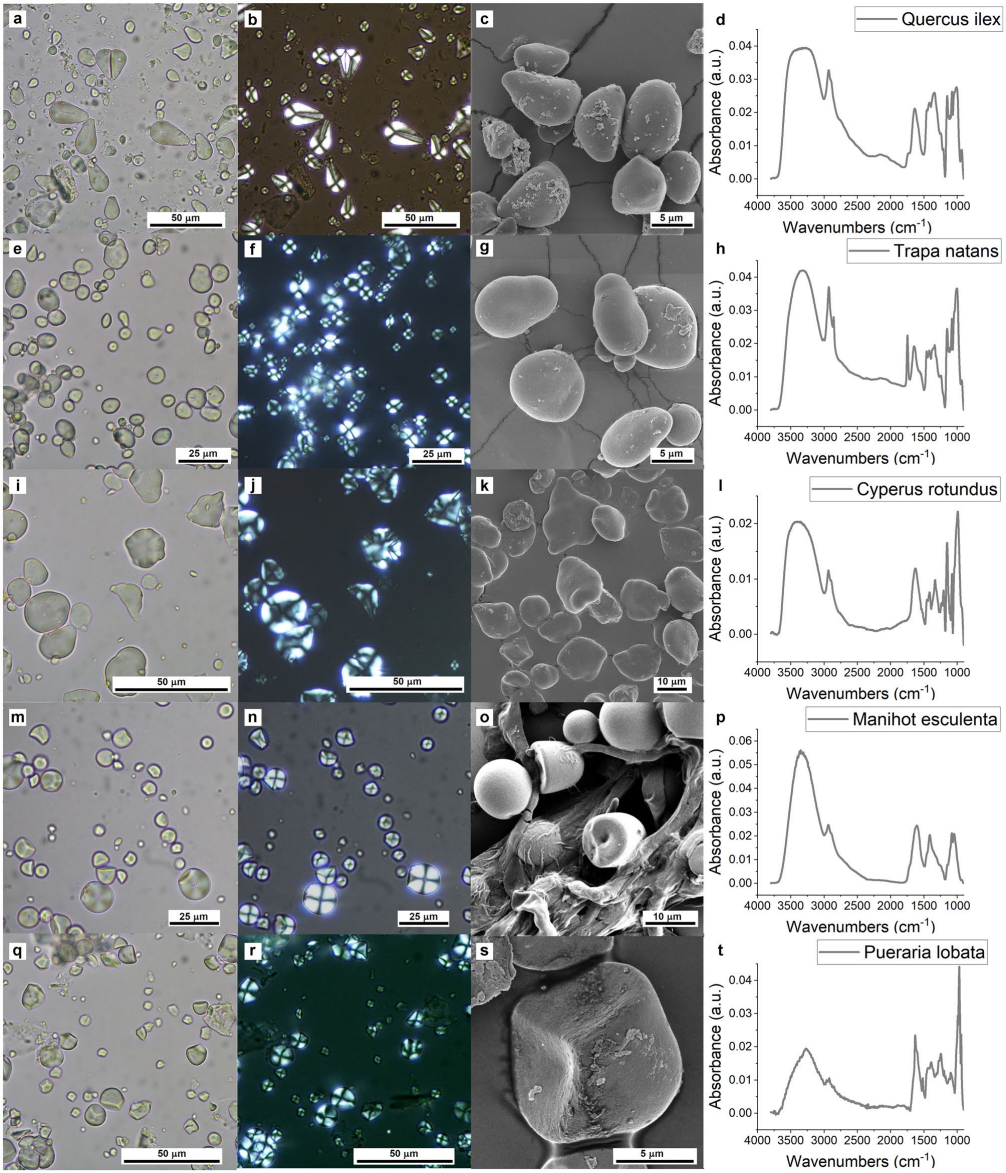
(**a, e, i, m, q**) (1st column) BF-OM, (**b, f, j, n, r**) (2nd column) P-OM, (**c, g, k, o, s**) (3rd column) SEM and (**d, h, l, p, t**) (4th column) FTIRM characterization of the modern starch references, ASO: *Quercus ilex*, *Trapa natans*, USO: *Cyperus rotundus*, *Manihot esculenta*, *Pueraria lobata*, from the 1st to the 5th rows.

In Fig. 1, OM bright field, BF (first column) and polarized, P (second column), as well as SEM images (third column) of selected MSRs are presented. The analysis of BF-OM images clearly highlights the large variety in shape and size of starch grains, while P-OM evidences the birefringence that is due to the almost crystalline pattern of α-glucans organization, which forms rings of amylose or amylopectin layered around the starch nucleation center called the hilum, resulting in the appearance of the Maltese cross, with the crossing point located at the hilum^7,8^.

From a chemical point of view, starch is a semicrystalline polymer, mainly composed of two polysaccharides: amylose and amylopectin. While amylose is mostly a linear chain of glucose molecules interconnected primarily by α-1,4 glycosidic linkages, amylopectin is a branched polymer with α-1,6 bridges that serve as branching points. The relative proportion among amylose and amylopectin in starches, as well as the specificities in the minimal amounts of lipids, in the form of Free Fatty Acids (FFAs) and Lysophospholipids (LPLs), as well as of proteins and minerals of which they are composed, are at the basis of the infrared classification of starches. In Fig. 1, fourth column, examples of MSR FTIR micro-spectra in the MidIR region (4000–800 cm^−1^) are shown. While it is beyond the scope of this paper to detail on the spectral attribution (which may be found elsewhere^25,26^), it does seek to underpin the most relevant information useful for ASC spectroscopic analysis. On this respect, three distinctive spectral regions can be identified: i- 3600–3100 cm^−1^ interval, for the –OH stretching, diagnostic for water content in starches, as along with the spectral band of bending mode of water centered at about 1640 cm^−1^, the intensity of which increases with reduced starch crystallinity, linked also to the presence of hydroxyl groups that may undergo etherification or esterification, in time^27^; ii-3000–2800 cm^−1^ interval, for the -CH stretching of methyl and methylene groups, the spectral shape of which correlates with the relative content of the two α-glucans and the possible presence of FFAs and LPLs; iii- 1500–800 cm^−1^, the fingerprint region associated to the vibrations of the glucose monomers of amylose and amylopectin (C–O, C–C, C–OH and CH_2_ bending modes), the one most diagnostic for the presence of starch^25^. As a matter of fact, the spectra variability among the selected MSR is more evident, as well as quite pronounced, in this latter spectral range.

### SEM characterization of SU-RBRs ASC-1, ASC-2 and ASC-3

**Figure 2:**
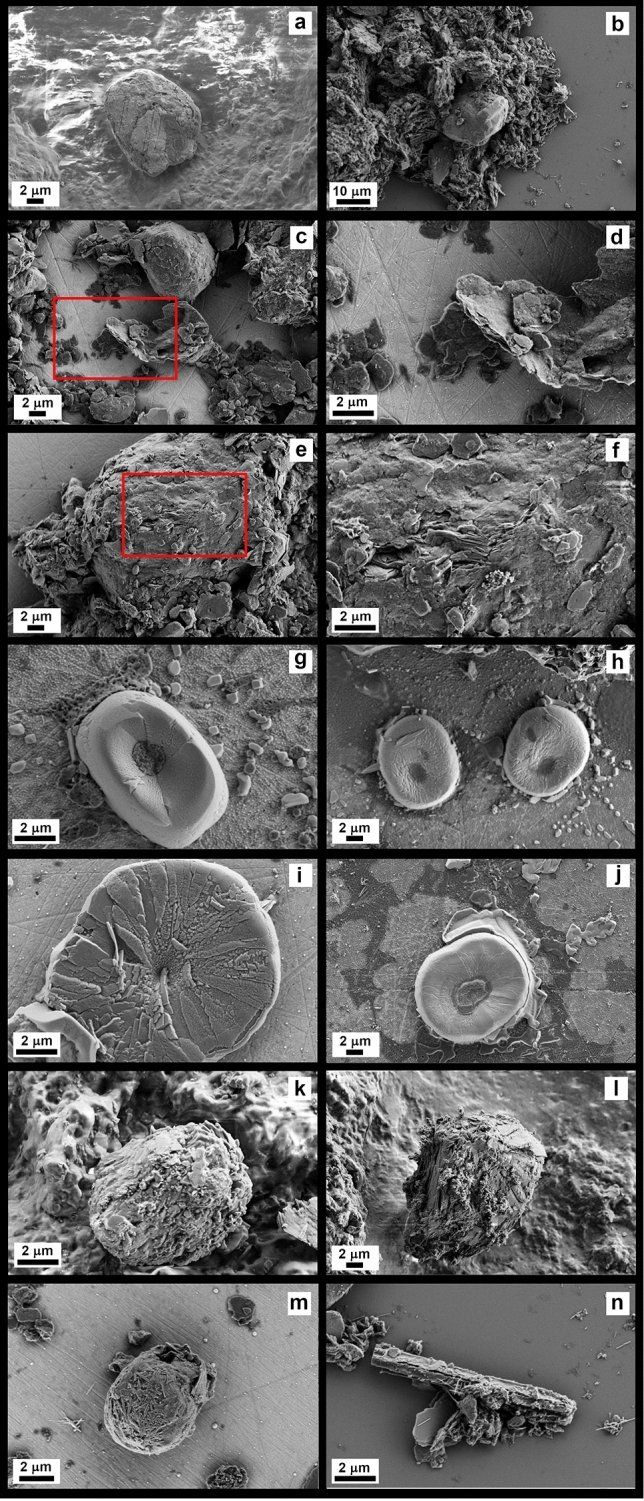
SEM characterization of the ASCs and SU-RBRs. SEM characterization of the SU-RBRs: ASCs and plant remains. SEM micrographs of starch grains extracted from different ground stones. (**a**) Brînzeni I tool # 833, ASC-1. (**b**–**f**) Brînzeni I tool # 833, ASC-2. Red rectangles in panels **c** and **e** defined the magnified regions in panels (**d**) and (**f**) respectively. (**g**–**j**) Surein I, large grinding stone, ASC-3. (**k**, **l**) ASC-1 from Kostenki 14-Markina Gora, tool #35. (**m**, **n**) Raphides surrounding a starch particle and the phytolith from Brînzeni I.

Figure 2 shows SEM images of ASC-1 to ASC-3 identified on ground stones from Brînzeni I tool # 833 (panels a-f), Surein I, large grinding stone #NN (panels g-j), and Kostenki 14-Markina Gora, tool #35 (panels k-l). Direct SEM of the molds (ASC-1) showed some particles, heavily surrounded by sediment, that may be tentatively identified as starch grains. The rounded particle shown in Fig. 2a is also associated to an area on the GST displaying some use related striations, interpreted as the result of mechanical pounding/grinding, likely hinting to an intentional processing of starch-rich storage organs performed by the large fragment of pestle #833 from Brînzeni I. In Fig. 2k,l, two ASC-1 from Kostenki 14 (#35, a broken pebble from layer III) are shown, and starch grains still adhering onto the mold’s crevices can be seen.

A large number of ASC-2 were identified in the Brînzeni I samples, dislodged from two GSTs (Fig. 2b–f). These particles are still surrounded by a significant amount of sediment; nonetheless they are still recognizable as starch grains by their polyhedral or rounded shape. Sometimes, their surfaces are not as smooth as in the case of the modern starches (See Fig. 1 for comparison), possibly due not only to sediment deposits, still adhering onto their surface, but also to different kinds of degradation and aging events. Some other ASC-2 grains from Brînzeni I show cracking, and exfoliation of the external surface and the typical internal amylose-amylopectin lamellar structure is evident (Fig. 2c–f).

ASC-3 type starches shown in Fig. 2g-j belong to the Surein I large grinding stone. According to this object’s biography^3,6,19^ (See Supplementary Material), the grinding stone was already thoroughly cleaned, hence the sonication allowed for the retrieval of few starch grains with less soil residues. Consequently, Surein I starch grains show a smoother surface when compared with the ASC-2 from Brînzeni I. SEM of the extracted Surein I starches highlights that they are mainly characterized by an oval or roundish shape and, in some cases, they show a radial fibrillary fractured surface, typical of crushed starch grains^28^. A peculiar conical crater was also observed in most of the Surein I starch grains.

SEM images also revealed the presence of other SU-RBR of vegetal origin diverse from starch gains (Fig. 2m,n). Among them we identified raphides, or needle-shaped oxalate crystals^28–30^. The co-occurrence of starch grains together with other plant elements, e.g., phytoliths, raphides, fibers, and parenchyma, strengthens the hypothesis that these GSTs were used to process plant material, an argument already put forward by Hardy B.L. and colleagues^31^ following their research on the flakes from the MIS 3 sites of Starosele and Buran Kaya III in Crimea. These evidences also lessen the probability that the presence of starch grains can be ascribed as the result of modern contamination.

### FTIR Imaging of ASC-2 and ASC-3 samples

**Figure 3:**
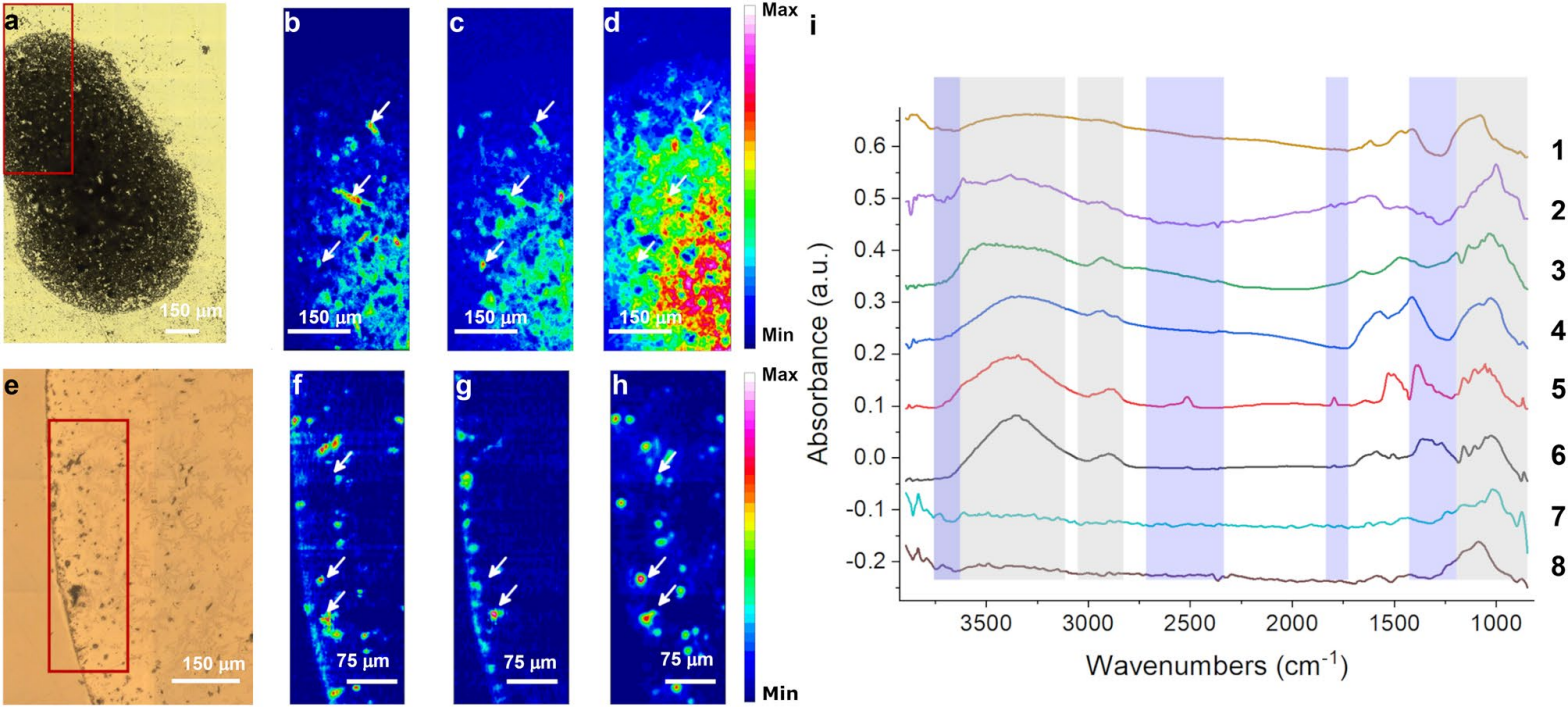
FTIR images of the ASCs. (**a**) Overview of a deposit of particles obtained from a GST fragment from Brînzeni I, BZ #NN; the red rectangle indicates the imaged area; the scale bar is 150 microns. (**b**–**d**). Heat chemical maps generated by the integration of specific ROIs: (**b**) – ROI1, (**c**) – ROI2, (**d**)—ROI3, the scale bars are 150 microns. (**e**) Overview of a drop of the suspension obtained by the sonication of mold n. 3 from Surein I, #NN; the red rectangle identifies the imaged area, the scale bar is 150 microns. (**f–h**). Heat chemical maps generated by the integration of specific ROIs: (**f**) ROI1, (**g**) ROI2, (**h**) ROI3 the scale bars are 75 microns. The hot spots (red–purple) represent the pixels where the integral value is higher; dark blue areas correspond always to zero. (**i**) Eight representative spectra of ASC-2 and ASC-3: **1** from Surein I, **2** to **6** from Brînzeni I, and **7**, **8** from Kostenki 14. The gray areas represent the spectral ranges more characteristic for carbohydrates, while the blue ones are those more typical for carbonate/mineral signals.

In order to go beyond the morpho-descriptive approach and to attempt the chemical characterization of the retrieved particles, the experimental plan was integrated with the label-free, non-damaging chemical characterization of the samples by using FTIRI at micrometric lateral resolution. More than 300 images were collected and processed from the ASCs extracted from the archaeological GSTs, and some of the results on select samples are shown in Fig. 3. In detail, optical images in panels 3a and 3e show an ASC-2 sample obtained from the Brînzeni I pestle BZ #833 and an ASC-3 sample from the Surein I grinding stone, respectively. It has already been highlighted that ASC-3 samples are far cleaner with respect to ASC-2 ones, and, as expected, round, starch-like objects may be recognized more clearly in Fig. 3e than in Fig. 3a. Whether the chemical profile of the identified particles and particle clusters points back to the one of a carbohydrate-base material is the reason for carrying out an FTIR analysis^25,32^.

According to the spectral features of the MSR previously described, which aimed at identifying ASCs, FTIR chemical images were generated by integrating the infrared hyperspectral data collected from specific regions of interest, ROIs, indicative of carbohydrates: ROI1, 3600–3100 cm^−1^ for the OH stretching (Fig. 3b,f), ROI2, 3000–2800 cm^−1^ for the -CH stretching of methyl and methylene groups (Fig. 3c,g), and ROI3, 1200–900 cm^−1^ for the carbohydrate backbone (Fig. 3d,h). As already highlighted, ROI3 is the most distinctive for carbohydrates. Nevertheless, at the same energies there can be a spectral interference due to the IR signals from metal-oxides^33^, silicates^34^, phosphates^35^ and sulfates^36^ putatively deriving from the soil both surrounding and/or adhering to the ASC^33^, as clearly revealed by the SEM images taken of ASC-2 and -3 samples. The occurrence of this cross-contamination generates “false” hotspots in the ROI3 chemical maps: as can be appreciated by observing the chemical images in Fig. 3d,h, it is clear how they portray all possible ASC hotspots, including those having a starch-like morphology, as well as minerals from the soil. In particular, in the deposition from Brînzeni I, the particles are so densely packed together, making it difficult to identify the starches, individually or in small groups, both in the BF-OM and ROI3 chemical image (Fig. 3d). Nevertheless, by comparing the same pixels in the other two chemical maps (Fig. 3b,c), it is possible to identify areas with common local maxima, indicated by the arrows, where the other peculiar carbohydrate signals are also intense, and therefore meaningful to identify ASCs. This reasoning is even clearer for ASC-3 samples from Surein I, which are less prone to soil-induced interference.

In accordance with the previously described approach, fifty chemical images from the collected 300 contained interesting spots that resulted in the identification of twenty-seven ASCs (as single-particles with an average diameter of a few tens of microns as well as small aggregates). Average spectra of ASCs were extracted from the hot-spots and some of them are presented in Fig. 3i. It must be highlighted that, in order to differentiate the ASC chemical profile from the surrounding soil from ASC-2, the spectrum of the surrounding material has been carefully subtracted, as described in Supplementary Fig. 2, where an example of the raw data, of the spectrum of the surrounding material and of the resulting subtraction can be seen.

**Figure 4:**
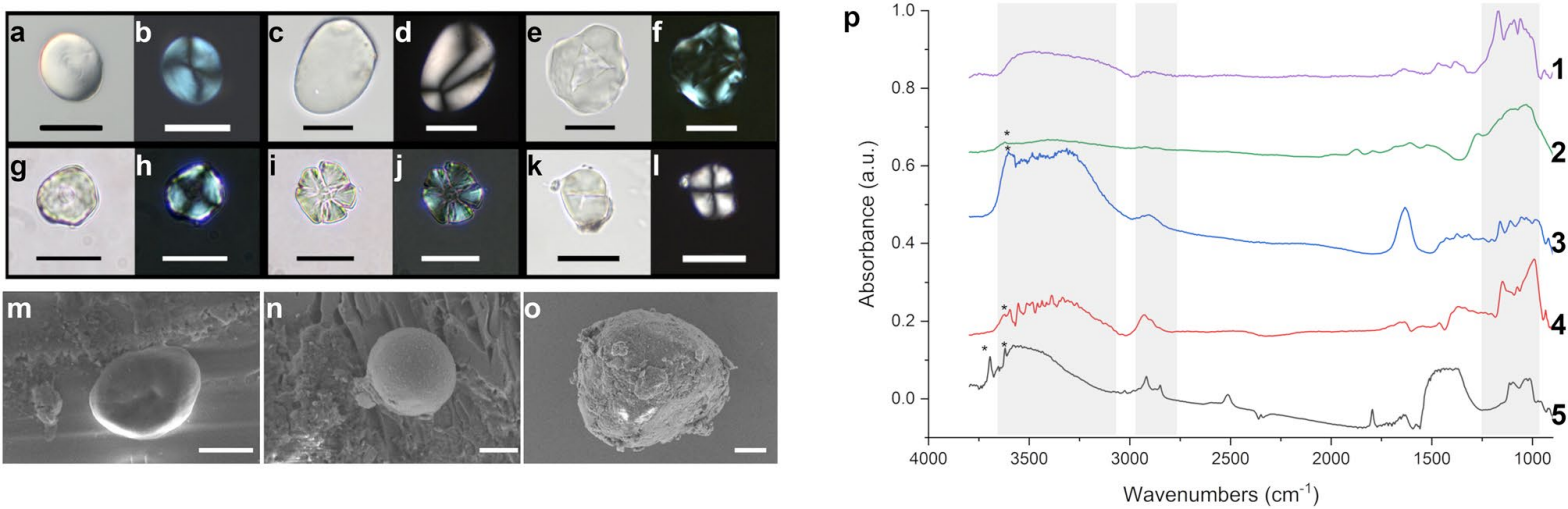
(**a**–**j**) Starch grains from Brînzeni I and Surein I under OM direct and polarized light. Starches from Brînzeni I: (**a**–**b**) #NN, (**c**, **d**) #177, (**e**, **f**) #833, (**g**–**h**) #3539, (**i**, **j**) #6707. Starches from Surein I: (**k**, **l**). All scale bars are 20 microns. (**m**–**o**) SEM micrograph of an ASC-4 from BZ#177, BZ#2965 and BZ#3539 respectively. Scale bars (**m**, **n**) 2 microns, (**o**) 5 microns. (**p**) IRSR-FTIRM spectra of some of the ASC-4 from: **1** BZ #6707, **2** BZ#833, **3** BZ#3539, **4** BZ#6707 and **5** BZ#442. The gray areas represent the spectral ranges more characteristic for carbohydrates signals.

**Figure 5:**
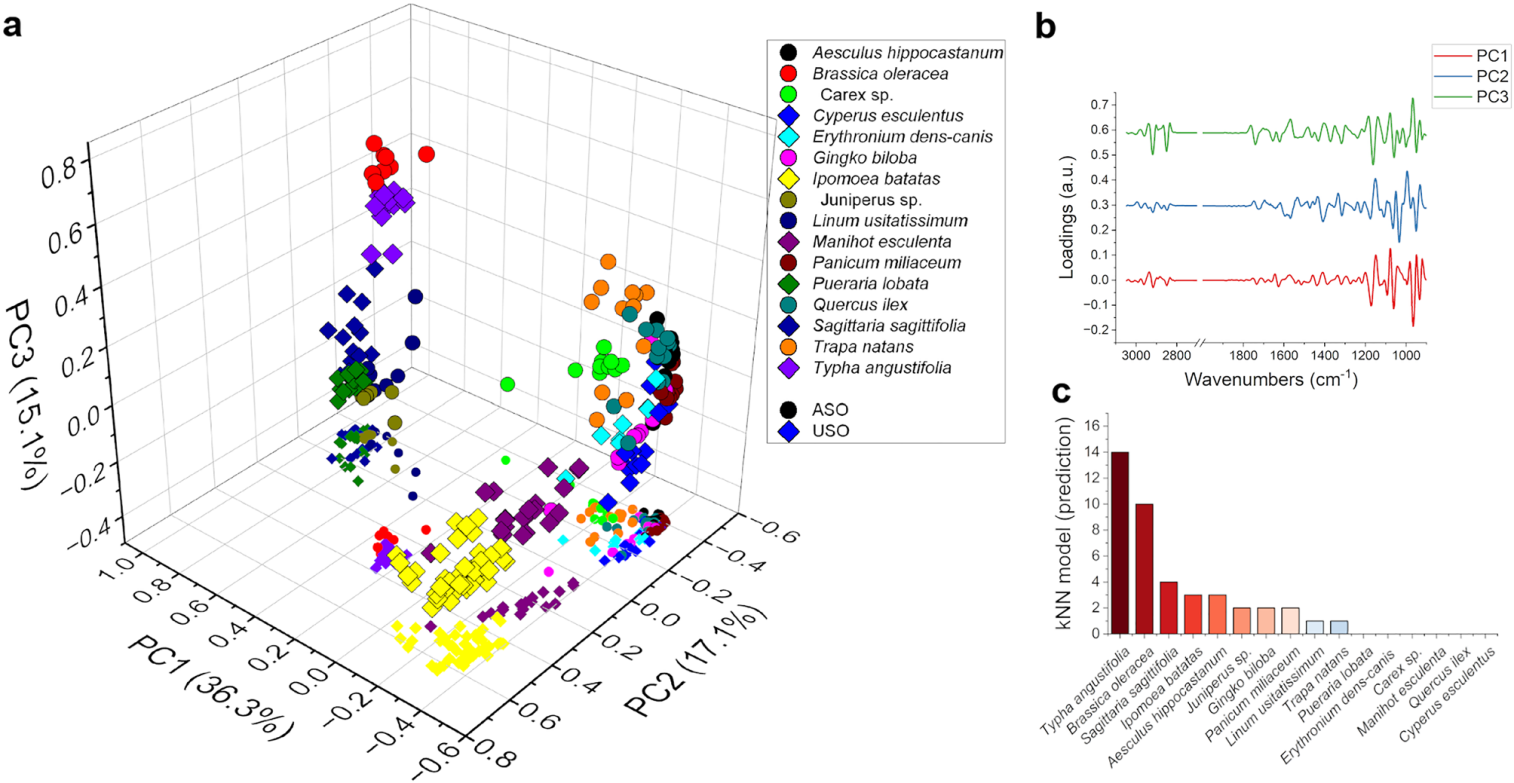
Chemometric model developed for ASCs classification. (**a**) Scatterplot of the MSR (spheres) and ASC (squares) in the PC1-PC2-PC3 space. (**b**) Spectral components representing PC1, in red, PC2 in blue and PC3, in green, in the spectra range 1800–900 cm^−1^. The spectra have been offset of 0.15 a.u. for clarity. (**c**) Bar chart representing the output of the classification model for all the ASCs measured. The highest clustering is within the USOs (roots, tubers, and rhizomes), compatible with the presence of resources across the Pontic Steppe.

ASC spectra in Fig. 3i have a degree of similarity with MSR spectra in Fig. 1, fourth column, that span from very similar (spectra 4, 6) to those barely recognizable as carbohydrates (spectra 7,8). Due to the large variability in the spectral profiles characterizing also modern starches, as previously highlighted, for spectra 1–6 in Fig. 3i, at this stage it is safer to comment only on the medium to strong –OH signals above 3000 cm^−1^ and weak to medium –CH stretching signals, with the main peak of the methylene moieties centered between 2920 and 2925 cm^−1^. Both signals may be a possible marker of a different level of degradation of the polysaccharides encompassing dehydration/dehydroxylation and the loss of peripheral polysaccharide chains. For Kostenki 14 ASCs (spectra 7, 8), the –OH signals above 3000 cm^−1^ and the –CH stretching signals in ROIs 1 and 2 respectively, are barely recognizable. The low intensity is also ascribable to the very small dimension (average diameter of a few microns) and non-aggregation of these starches, that justify the poorer spectral quality. For the latter samples, the spectral profile of ROI3 is silicate-like, while for sample 1 to 6 it is more complex and possibly deriving from the band overlap of minerals and ring vibrations characteristic of glycosidic linkage and sugar rings. In addition, FTIR micro-spectra in Fig. 3i-5,6 present signals from calcium carbonate, like the broad intense band at ~ 1430 cm^−1^, the C=O stretching at 1790 cm^−1^ and the overtone at 2520 cm^−1^ ^37^. Some clay-like signals at higher frequencies, with sharp peaks at 3612–3614 cm^−1^, can be seen in Fig. 3i-2,3. The same spectra exhibit also a shoulder at ~ 1322 cm^−1^, possibly assignable to calcium oxalate crystals^38,39^. These features could not be smoothed by subtracting the contribution of the surrounding materials; therefore, it is possible to hypothesize that they are not due to soil contamination, but may be indicative of the aging associated to mineralization, partial or complete, of the ASCs. In order to prove this hypothesis, an isolation procedure of the starches compatible with FTIR analysis was developed to obtain ASC-4, as already described in^6,19^, and exploited for further and finer IRSR FTIRM analysis.

#### OM microscopy, SEM and IRSR FTIRM of ASC-4 sample

A total of 111 starch grains (type ASC-4) have been identified by conventional OM inspection on the GSTs from Brînzeni I (n = 100), Surein I (n = 7) and Kostenki 14-Markina Gora (n = 4). Some optical images of recovered ASC-4 starches (both BF-OM and P-OM) are shown in Fig. 4. The size of the grains is micrometric, averaging less than 50 μm, hence sitting in the smaller size-range of the distribution of the most common starches. Despite the fact that botanical identification proved to be difficult because of the relatively poor conservation of the large majority of the starch grains, but also due to a lack of a reference collection of wild taxa consistent with the geographic region and the relative time period^40,41^, it was possible to distinguish different morphologies and to detail diagnostic features, namely the hila and lamellae that made it possible to identify them as starch grains. The Brînzeni I starches bear a wider variety of morphologies, appreciable thanks to the larger quantity of starch grains (one of the largest ever reported from the Early Upper Paleolithic—EUP), which include lenticular (Fig. 4a,b), polyhedral (Fig. 4e,f), and roundish starch grains (Fig. 4 g-h) and some that can be identified as probably belonging to USOs^42,43^ (Fig. 4c,d). From Brînzeni I again, polyhedral grains, which range in size between 15 and 23 µm, were observed (Fig. 4 i,j), and from Surein I some more oval grains were recovered, about 20 µm wide (Fig. 4k,l). Damages were evident on a large majority of the starch grains from the three archeological sites and include broken or crushed grains (Fig. 4 i,j), deformed grains (Fig. 4k,l), circular or uneven depressions affecting the central parts of the grains where the hilum is located, associated with the loss of definition of the extinction cross, possibly due to pounding and/or gelatinization (Fig. 4e,f). These damages suggest different types of mechanical forces or processing activities, or even taphonomic processes^44–47^, while pits and cavities are interpreted as the result of starch biochemical degradation, by soil enzymes or other biogenic agents like fungi or bacteria. The SEM images serve to present more structural details, but also to visualize the smaller starch grains which would be barely visible using the OM. This is especially true in cases such as Fig. 4 m–n, where the elements are smaller than 10 microns.

In order to characterize the vibrational profile of the isolated ASCs previously characterized by P-OM, IRSR FTIRM was carried out on the starch grains. In Fig. 4p are shown the FTIR spectra of five ASC-4 from Brînzeni I (#442, #6707, #833 and #3539). All spectra exhibit peculiar carbohydrate features, associated to –OH stretching, –CH stretching and ring vibrations in the spectral region 1200–900 cm^−1^, whereas relative intensity and positions of the bands can vary sample by sample, as already highlighted for ASC-2 and ASC-3. Isolated ASC-4 have negligible traces of soil residues and contaminants that could alter both their infrared spectrum and morphology. Therefore, since the main spectral features of ASC-4 are comparable to the ones of ASC-2 (after soil background subtraction, supplementary Fig. 6) and of ASC-3 (Fig. 3i), we can therefore confirm the spectral attributions done on ASC-2 and ASC-3 datasets and the hypotheses of their partial mineralization. This statement is more clearly verifiable for BZ #442 (Fig. 4p_5, black line), the spectrum of which is characterized by distinctive CaCO_3_ signals, already commented. It must be highlighted that the carbonate stretching band is so intense as to be saturated, therefore allowing the clear view of the other weaker carbonate spectral features. In addition, the FTIR spectra of the starch isolates from BZ #442 present an overall lower intensity in the 1200–900 cm^−1^ spectral region, and very sharp methylene peaks (at 2920 and 2850 cm^−1^). Since P-OM and SEM undoubtedly identify these particles as starches, no doubts can be raised on their polysaccharide nature, while the mineralization of the starch, as a consequence of diagenetic processes, led to the deposition of carbonate minerals still preserving the starch original structure. In addition, the presence of sharp absorptions in the spectral range 3750–3580 cm^−1^, marked with an asterisk (*) in Fig. 4, usually attributed to highly ordered water molecules in kaolinite, let to postulate also a partial silicification of the starch particles. The starch from BZ#3539 (Fig.p_3, blue line), which is quite high in water content, and from BZ #6707 (Fig. 4 p_4, red line) both retain distinctive starch microfossil traits, despite being less evident than sample BZ #442.

Any other band attribution and speculation would not be reliable and indeed not verifiable since there are no chemical complementary characterization tools that may work at the single starch level to support the band attribution. Nevertheless, ASC-2 to ASC-4 analysis allows to conclude that the spectral profile of ancient starches differs from their modern counterparts, being characterized by mineral features, indicative of the diagenetic process they underwent and support the attribution of the analyzed starch candidates as being genuine (ancient).

### ASC classification on MSR model

In order to attempt a classification of the identified genuine ASCs, the acquired data on MSR were used to build a model suitable for categorizing the ASC, as specified in the Methods section. In Fig. 5a the (PC1, PC2, PC3) MSR score-plot is shown. The scores of each MSR replicate cluster together, proving that the spectral variability within the same plant is lower than the one between the different plants, and therefore allowing for a good distinction between them. In Fig. 5b are shown the spectral loadings of PC1, PC2 and PC3, representing the ensemble of the spectral features that account for the largest variance for each component. PC1 main peaks are at 1135 cm^−1^, from the C–O–C asymmetric stretching, 1065 cm^−1^ and 985 cm^−1^ usually assigned to non-structural carbohydrates^26,48^. PC2 has spectral features similar to those of PC1 at low wavenumbers, with the main peaks at 1174 cm^−1^ from ring “breathing” vibration, 1065 cm^−1^ due to in-plane bending of C–OH bond and 1033 cm^−1^ assigned to the C–OH stretching^49^. At higher wavenumbers, their strong peak at 1428 cm^−1^ from β-glucans can be seen. PC3 presents strong signals at 1163 cm^−1^, 1060 cm^−1^ and 950, similarly to PC1 and a peak at 1740 cm^−1^, assigned to the C=O stretching of aldehyde group possibly from galacturonans^50^, and 1658 and 1622 cm^−1^ signals from proteins. In the high wavenumber region (3100–2800 cm^−1^) PC3 presents sharp peaks from CH_2_, probably due to the polysaccharide’s backbone or lipid aliphatic chains.

Despite the 1500–800 cm^−1^ spectral region confirmed to be the most diagnostic for the presence of starch and, at the same time, the mostly affected by starch mineralization, the application of the identification model to the unknown ASCs performed quite well, given that all the performance indicators are higher than 0.9 (see Methods for more details) and allowed to assign to the ASCs a degree of similarity to the starch-rich organs from which they were extracted. As can be appreciated from the bar chart in Fig. 5c, the majority of the tested starches are similar to those derived from rhizomes and tubers (e.g., USOs). It is worth noting that, for example, the mineralized BZ#442 starches could also be classified as originating from a rhizome, with an 80% or higher accuracy.

While this analysis is not intended to be conclusive, it does prove that ancient starch identification can be attempted at the individual starch level based on an IRSR FTIRM model built on MSRs. The model must certainly be further expanded and, to increase the classification precision, it must be optimized on real train datasets of already recognized ancient starches, if any, or their closest approximation, i.e., artificially aged ones. We are working on addressing this later point in future research.

## Conclusions

To date, the direct evidence of starch-rich plant processing during the Late Pleistocene (MIS 3) has been questioned due to the inability of optical microscopy imaging techniques to confirm their genuine ancient origin. Our method, which combines imaging techniques such as optical and polarized light microscopy with scanning electron microscopy (SEM), and chemo-profiling using FTIR spectral imaging, achieves a chemical sensitivity enhanced by the superior brilliance of IRSR, allowing us to directly analyze individual starch grains.

By employing it we could observe more detailed morphological features of the starch grains, such as their lamellar structure, as well as signs of mechanical processing, including, fractures, cavities, and exfoliation. Through the combination of OM, SEM, and FTIR analyses, we have achieved a groundbreaking milestone: the identification of specific chemical characteristics at the individual starch grain level, which distinguish the ancient starches extracted from ground stones dating back to MIS 3 with respect to the modern counterpart.

The FTIR analysis is particularly valuable as it revealed strong bands associated with mineralized material. This enables us to differentiate between modern and ancient starches, even in complex matrices. Our approach provides evidence that the effects of aging associated with the biomineralization of starches could be observed in ASC-2 and ASC-3-type samples. Despite the mineralization features of ASCs mostly affect the 1500–800 cm^−1^ spectral region, which is also the more diagnostic of the starch botanical origin, we could classify the retrieved ASCs using a model built on MSRs as largely originating from underground storage organs (rhizomes and tubers). The chemically characterized ASC-4, which consists of isolated and purified starches from various MIS 3 sites (Kostenki 14, Surein I, and Brînzeni I), still display the characteristic Maltese cross. This, along with the observed mineralization features, strongly supports the distinctiveness of genuine ancient starches. These findings are in line with the data obtained from the mineralized starch grain measured in BZ#442, which can be interpreted as originating from a rhizome with an accuracy rate of at least 80% (or higher).

It is important to note that FTIR imaging and microscopy with IRSR operates at the individual starch grain level. This unambiguous association is a prerequisite for coupling chemo-profiling with OM and SEM. Our ability to identify single starch grains confidently helps us avoid two issues: i) confusing a genuine ancient starch with a modern one due to their different vibrational profiles, and ii) misclassifying roundish objects that show the Maltese cross as starches. Furthermore, our data has been crucial in identifying the origin of the archaeological starches, primarily as originating from underground storage organs (USOs). However, more detailed taxonomic attribution remains challenging due to the limited number of plant species available in the reference collection. Expanding the reference collection to include more wild plant species is a future goal.

Overall, our research yields several significant findings. Firstly, it provides definitive answers to long-standing questions regarding the preservation and detectability of starch over millennia. Secondly, our ability to identify the genuine origin of starch found on ground stone tools from MIS 3 offers crucial evidence to support their attribution. Thirdly, our study demonstrates the feasibility of studying structured use-related biogenic residues from museum collections, providing a reliable sampling approach for future analyses. Furthermore, our study holds significance in understanding the evolutionary success of modern humans. The secure identification of genuine ancient starches, still detectable on ground stone tools, allows us to reconstruct the complex processing techniques of early modern humans exploiting diverse foodscapes. This processing included the consumption of dietary carbohydrates primarily obtained from underground storage organs (USOs). Our results strongly support the notion that starch rich food processing played a vital role in providing highly calorific nutrients during the early colonization of the West Eurasian boreal latitudes, dating back at least 40,000 years.

## Materials and methods

### Materials

The examined ancient starch grains were recovered from the used surfaces of nine ground stones, preliminarily identified during museum collection using low magnification stereomicroscope with magnification up to 60X. The GSTs were retrieved from three sites located across the Pontic Euxine steppe: Brînzeni I (Moldova), Surein I (Crimea), and Kostenki 14-Markina Gora (Don River), according to Table 1. Detailed information on the GSTs can be found in the Supplementary Materials and they are summarized in Table 2.

Modern Starch Reference (MSR) were prepared by grinding 5 g (or multiple aliquots) of each of the selected raw storage organs by means of a blender and the chopped residue was soaked in 200 mL of ultrapure water (with a resistivity of 18.2 MΩcm, obtained with the Milli-Q® system Millipore) for 3 h. The mixture was then filtered off in a Millipore filtration apparatus (Merck Millipore Glass Vacuum filtration system) by using a metallic filter with pore size of 0.1 mm. The filtered water containing the starches was then centrifuged (Jouan CR3i centrifuge, UK) at a Relative Centrifugal Force (RCF) of 6147 for 5 min at 20 °C. The pellet, obtained by removing the supernatant, was washed with 100 mL of ultrapure water and the mixture was bath sonicated for 5 min before recovering the pellet by further centrifugation (same conditions as above). The precipitated matrix was then washed with 5 mL of EtOH (Sigma Aldrich), followed by the sonication and centrifugation processes as described above and the EtOH was removed. The pellets recovered after this extraction procedure were dried and the powders obtained were stored under dark conditions.

### Methods

#### Optical microscopy, OM

The archaeological samples were processed in two different institutions (CNRS in Nanterre (France) and IHAE FEB RAS in Vladivostok, Russia) and observed with different optical microscopes with both unpolarized and polarized light (Nanterre: optical microscope Nikon E600 POL Eclipse, magnification between 100 and 600×; Vladivostok: ZEISS Axioscope A1 up to 800×). The MSR were collected, prepared and extracted at DAIS in Venice and the OM images were visualized at the ArchéoScopie Platform of the *MSH Mondes* in Nanterre (France) while the SEM samples were viewed at the Service de Microscopie Électronique (SME) at the *Institut de Biologie Paris-Seine* using a high-resolution SEM (GeminiSEM 500, Zeiss). Optical images were also collected with the Vis-IR Hyperion 3000 microscope (see section *FTIR Imaging and Microscopy, FTIRI and FTIRM*).

#### Scanning electron microscopy, SEM

In accordance with Table 2, both archaeological and modern samples were investigated at IOM-CNR (Trieste) with Zeiss Supra 40 high resolution Field Emission Gun (FEG) SEM with 3rd generation Gemini column, equipped with Everhart–Thornley Secondary Electron Detector and a High efficiency In-lens detector, the latter providing an increased signal-to-noise ratio in image acquisition. Both ASC and MSR starch grains were imaged without coating and observed with FEG modality at low voltage 3–5 kV and a 4pA–10nA probe current reaching even 25,000×magnification. The SEM used is the same one mentioned above (section *Optical Microscopy, OM*).

Droplets of 0.05 mL were deposited on ZnSe windows, left to dry, and scanned uncoated to guarantee the reliability of the complementary analytical steps, like FTIR and possibly other analytical techniques (e.g., Raman microscopy).

#### FTIR imaging and microscopy, FTIRI and FTIRM

ASC-2 to ASC-4 and MSR samples (as detailed in Table 2) were measured at SISSI-Bio (Chemical and Life Science Branch of Synchrotron Infrared Source for Spectroscopy and Imaging beamline) at Elettra – Sincrotrone Trieste using a Bruker Hyperion 3000 Vis-IR microscope coupled with a Bruker Vertex 70 V interferometer^51^. FTIRI with transmission sampling geometry was applied on ASC-2 and ASC-3 samples: the microscope is equipped with a 64 × 64 pixel Focal Plane Array (FPA) detector capable of acquiring a full FTIR spectrum per pixel, thus generating 4096 pixels’ hyperspectral images for each measure. Given the 15× magnification of the used Cassegrain objective, the pixel size is about 2.6 × 2.6 microns and the field of view of one image tile is about 167 × 167 microns. Mosaics containing multiple tiles were also acquired for each sample. The instrumental parameters used for FTIRI measurements were the following: 64 scans at 8 cm^−1^ spectral resolution, 5 kHz scanner speed. More than 300 hyperspectral maps were collected. Isolated ASC-4 starches were measured with the same instrument by FTIRM in transmission sampling geometry, using Infrared Synchrotron Radiation (IRSR) source and a Mercury Cadmium Telluride (MCT-A) detector. The parameters used for each IRSR measurement were the following: 512 scans at 4 cm^−1^ spectral resolution, 120 kHz scanner speed, setting the apertures at the same size of the starches, from 20 × 20 to 40 × 40 microns. For both FTIRI and IRSR FTIRM measurements, the background was collected on a clean portion of the window support, with the same parameters used for the sample data collection. For MSR, the spectra have been acquired with FTIRM with conventional source, averaging 128 scans for each spectrum at a spectral resolution of 4 cm^−1^ setting the scanner speed at 40 kHz. With the MSR being much more abundant than ASCs and being deposited in a densely packed layer, the lateral resolution was set at 50 × 50 microns.

Both FTIRI and FTIRM data were analyzed in OPUS 8.5 (Bruker Optics) and Quasar (https://quasar.codes)^52,53^. FTIRI and FTIRM data were pre-processed by applying the water–vapor compensation routine of the proprietary Bruker software OPUS 8.5. Due to spectral interference from surrounding material, of mineral origin mainly from the soil, ASC-2 spectra had to be further pre-processed before interpretation by subtracting this contribution: the spectrum of the surrounding material was obtained by extracting an average spectrum from pixels adjacent to the target particle. More details on the method are reported in the caption of Supplementary Fig. 2.

### Chemometric model

A chemometric model was built using MSR spectra as training dataset. The model was developed in Quasar (https://quasar.codes). MSR were preprocessed as follows: interpolation to a common spectral range and data spacing, baseline correction with a rubber band method, derivatization using the Savitzky-Golay algorithm set at 23 window points 3rd degree polynomial and 2nd grade derivative, and vector normalization. This data was then reduced using a principal component analysis (PCA) to 16 principal components that represent the 95% of the total variance. Within this 16-dimensional PCA space, k-Nearest Neighbor (kNN) algorithm was used with 4 points and the weighting distance calculated as Euclidean distance. The performances of the obtained model were evaluated by stratified cross validation repeated 6 times. The performance indicators are the following:

- Area under the Curve (AUC): 0.990.
- Classification Accuracy (CA): 0.925.
- F-1 Parameter: 0.922.
- Precision: 0.927.
- Recall: 0.925.

### Supplementary Information

Supplementary Information.
