# Supplementary material for: Morpho-chemical characterization of individual ancient starches retrieved on ground stone tools from Palaeolithic sites in the Pontic steppe
Source: Sci Rep. 2023 Dec 7;13:21713. doi: 10.1038/s41598-023-46970-8 (PMC10709628; doi:10.1038/s41598-023-46970-8)
Supplement: Supplementary file 1 — Supplementary Information. [file 41598_2023_46970_MOESM1_ESM.docx]

**Supplementary Materials**

**Morpho-chemical characterization of individual ancient starches retrieved on ground stone tools from Palaeolithic sites in the Pontic steppe**

G. Birarda^1*^, E. Badetti^2*^, C. Cagnato^3,4*^, G. Sorrentino^5*^, I. Pantyukhina^6^, C. Stani^7^, S. Dal Zilio^8^, G. Khlopachev^9^, S. Covalenco^10^, T. Obada^11^, N. Skakun^12^, A. Sinitsyn^12^, V. Terekhina^9,12^, A. Marcomini^2^, C. Lubritto^13^, N. Cefarin^1^, L. Vaccari^1*^, L. Longo^2,14*^

***Affiliations***

^1^ Elettra-Sincrotrone Trieste, S.S. 14 - km 163,5 in AREA Science Park, 34149 Basovizza, Trieste Italy;

^2^ Ca' Foscari University of Venice, Department of Environmental Sciences, Informatics and Statistics, via Torino 155, 30172 Mestre (VE), Italy;

^3^ UMR 8096 Archéologie des Amériques; CNRS-Université Paris 1 - Panthéon-Sorbonne, France;

^4^ UMR7268 Anthropologie bio-culturelle, Droit, Ethique et Santé (ADES) Marseille, France;

^5^ Department of Physics, University of Turin, Italy;

^6^ Institute of History, Archaeology and Ethnology, Far-Eastern Branch, IHAE-FEB RAS Vladivostok, Russia;

^7^ CERIC-ERIC, S.S. 14 - km 163,5 in AREA Science Park, 34149 Basovizza, Trieste Italy;

^8^ CNR IOM, S.S. 14 - km 163,5 in AREA Science Park, 34149 Basovizza, Trieste Italy;

^9^ Peter the Great Museum of Anthropology and Ethnography (the Kunstkamera) of the Russian Academy of Science, St. Petersburg, Russia;

^10^ Institute of Cultural Heritage, Academy of Sciences of Moldova, Moldova;

^11^ Institute of Zoology, National Museum of Ethnography and Natural History of Moldova, Moldova;

^12^ Institute for the History of Material Culture, IHMC-RAS St. Petersburg, Russia;

^13^ Department of Environmental, Biological and Pharmaceutical Sciences and Technologies, University of Caserta “Luigi Vanvitelli”, Via Vivaldi 43, 81100 Caserta, Italy;

^14^ ADM School, Nanyang Technological University, Singapore

^1^*giovanni.birarda@elettra.eu

^2,14^*laura.longo@unive.it

L. Longo and G. Birarda provided the Supplementary Online Materials

G. Sorrentino edited Supplementary Fig. 1.

**Index**

1. **The geographical context of the research**
2. **Archaeological sites**

**2.1	Brînzeni I** (Moldova)

***2.1.1*** ***Brînzeni I: object’s biography***

**2.2	Surein I** (Crimea)

**2.2.1 *Surein I: object’s biography***

**2.3	Kostenki 14-Markina Gora** (Russia)

**2.3.1 *Kostenki 14-Markina Gora: object’s biography***

3. **Sample preparation**
4. **Spectra subtraction**
1. **The geographical context of the research**

Ancient starch candidates (ASCs) have been retrieved from stone tools used to grind and pound starch-rich organs by humans who lived across the Pontic steppe starting around 40 ka BP. The Pontic steppe covers the southwestern part of the Eurasian Steppe Belt, a very wide east-west plain crossed by a mosaic of river valleys from the Dnieper to the Volga Rivers expanding eastwards towards the Anuy River (Siberia), and by high plateaus from the Carpathians to the Urals until the Altai Mountains (Central Siberia). Since antiquity, the Pontic Steppe has been known as the *Pontus Euxinus* (Euxeinos Pontos), and covers the Mediterranean coastal areas overlooking the Black Sea and the Caspian Sea (Supplementary Fig. 1). It is a territory rich in caves and shelters, good quality raw materials, and it has plenty of animal herds; it also functioned as an open nexus between the northern and southern boreal territories. This biome is a rich steppe-like grassland dominated by shrubs (including large graminoids) with spots of forest-steppe and this biotic diversity provided a wide range of starch-rich plants throughout the year^1^. The area sets the scene for one of the dispersal routes into Western Eurasia by *Homo sapiens*, who most probably crossed paths and mated with local groups of late Neanderthals, still surviving in the southern refugia^2–4^. It is possible that small, mobile groups of hunter-gatherers strategically foraged on a broad spectrum of resources that includes starchy plants available in the rich biome of the Pontic steppe during the MIS 3.^5,6^ Within the periglacial loess-steppe environment of the East European Plain, the analyses are centered on the structured use-related biogenic residues (SU-RBR) dislodged from ground stone tools (GSTs) retrieved from 3 sites: Kostenki 14 – Markina Gora, Brînzeni I cave, and Surein I rockshelter (Supplementary Fig. 1).

**Supplementary Fig. 1**. Location of Kostenki 14 – Markina Gora, Brînzeni I, and Surein I sites across the Pontic steppe, on the northern rim of the Black Sea. On the right, examples of the GSTs involved in this study: one small grinding stone from Kostenki 14 - Markina-Gora, the fragmented pestle and grinding stone from Brînzeni I, and the large grinding stone from Surein I.

2. **Archaeological sites**
   2.1 **Brînzeni I**

The cave opens on the Racovăț River, a tributary of the Prut River, in the Edinet district, north-western Moldova. The Prut River area was one of the most densely occupied areas during the Upper Paleolithic - 32 and 27 ka uncal BP (36,000 and 30,000 years ago) - west of the Carpathians^7^. The Brînzeni cave, 17 x 14 x 4 m (maximum height) lays at 65 m asl and its mouth is oriented towards the north. Near the site, the Miocene limestone outcrops are rich in high quality flint, possibly one of the reasons for the successful presence of modern humans in the territory during the crucial time for the early colonization of south-eastern Europe.

The site is recorded as yielding one of the most relevant Upper Paleolithic assemblages within the so-called “Prut River Culture”^5^. Its rich lowermost “cultural layer III” has been attributed to Aurignacian^7^, therefore representing an early presence of *Homo sapiens* in the area (as supported by 3 recent AMS radiocarbon dating on bone collagen - Beta 462731, Beta 462732, Beta 462733 - carried out by the authors in 2017, unpublished data). The site was discovered by N.A. Chetraru – who systematically excavated the settlement over several campaigns (1963-65, 1968 and 1975 and by and I.A. Borziak in 1987) – and yielded extraordinary art works, among which is worth mentioning the ivory pendant or amulet (a definition that N. A. Chetraru preferred, 2006 personal communication) retrieved in the inner part of the cave, near the bottom of cultural layer III.

The area of the Prut River underwent severe climatic downturns during MIS 3 and the periglacial/steppe conditions supported by pollen analysis match with the occurrence of horse, reindeer, and bison, the large herbivores most targeted by Brînzeni I dwellers. Regarding the vegetal covering, trees are represented by *Pinus sylvestris*, *Betula*, *Ulmus*, *Tilia,* *Corylus* and *Picea*, with the presence of Poaceae, Chenopodiaceae, and Asteraceae, including *Artemisia*^7^. In 1987 S.I. Medyanic collected samples from different stratigraphic layers, for pollen and spore analysis. The study reveals the absence of artic-alpine and boreal elements and the presence of taxa compatible with wooded steppe surrounding the site. The pollen list available for Brinzeni I guided which plants to include in reference collection. Among the reported species we analyzed starch grains from taxa within different families: Alismataceae (water-plantains, *Sagittaria sagittifolia*), Brassicaceae (*Brassica oleracea*), Cupressaceae (*Juniperus* sp.), Cyperaceae (*Carex* sp. and *Cyperus esculentus*), Fabaceae (*Pueraria lobata*), Liliaceae (*Erythronium dens-canis*), Poaceae (*Panicum miliaceum*), Polygonaceae (*Polygonum aviculare*, *Fagopyrum* sp.), Sapindaceae (*Aesculus hippocastanum*), Typhaceae (*Typha angustifolia*). All reported plants include taxa with starch-rich storage organs (cambium for trees, nuts, rhizomes and seeds for grasses).

***2.2.1 Brînzeni I Object’s biography****.* Among the 114 pebble stones collected during the excavations, 36 were selected as putative ground stone tools and are currently under analysis from morphological, traceological, and residual perspectives. On the basis of their petrographic analysis, the stones can be attributed mainly to calcarenite and quartz-arenite, most likely collected from the Racovăț River that flows below the cave.^8^ We here report on the structured use-related biogenic residues (SU-RBR, see main text for definition) retrieved on five of the sampled GSTs: a pestle broken into two pieces, BZ #833 and BZ #2965; a reconstructed grinding stone (a lower base, still incomplete) composed of two fragments BZ #442 and BZ #NN (No Number); another large grinding stone, BZ #6707 and a smaller one BZ #3539, and a broken pestle BZ #177. Regarding the object biography^5,6,9^ the following information can be relevant to frame the research questions and to parsimoniously interpret the results of the analyses. According to Chetraru’s field notebooks available in the museum collections, the investigated ground stones were mapped on the site plan/grid during the excavation, roughly washed in the river near the site, and ink labeled; after these preliminary procedures they were stored in wooden boxes that are enclosed in metal cages and kept in the storage facilities of the National Museum of History of Moldova (NMHM, Chisinau, Moldova), where they were accessed by the authors in 2015 and 2016, under an overall research agreement between Nanyang Technological University, NTU, Singapore, and NMHM. The fact that many of the retrieved pebbles bear large aggregates of residual sediment, as well as some fragments of carbonate crusts adhering to the surfaces, supports the notion their handling was very limited before our visit. Moreover, two GSTs, namely a long pestle and a large grinding stone, were refit thanks to our analysis, in turn suggesting that the non-flaked assemblage was not handled prior to our study.

During the sampling campaigns, the stones were preliminary inspected with a stereomicroscope and a metallographic microscope (Olympus BHMJ metallographic microscope).^5,6^ Hence, putative used areas were identified by the damage patterns observed and then sampled for further analysis. A sketch of the flowchart describing the GSTs cleaning and U-RBRs retrieval is presented in Supplementary Figure 5. Two different strategies were followed for residue extraction from the identified used areas. The active areas of aa first group of GSTs (2016 campaign, #442, #NN, #833, #2965, #177) were sonicated, immersing the relevant areas in 100-200 ml of MQ® water and placed in an ultrasonic bath at the Chemistry Department of the State University of Moldova (Chişinău). The suspension obtained was centrifuged two times at 6000 rpm and the supernatant discarded in order to reduce the volume, transferred into 2 mL vials, where a few drops of Ethanol were added (Longo et al. 2021^5^, Fig. 3). During the successive campaign (2017) other stones were processed and here we present the analysis of #3539, #6707, two large grinding stones (passive tools). The active area of each stone was soaked in ultrapure water for 2 hours by keeping the beaker covered to avoid being contaminated. In this case neither the sonic bath nor the centrifuge were available. The solution was poured in two 50 ml tubes and a few drops of ethanol was added. The solution was then centrifuged at NTU lab facilities in Singapore and the obtained pellet was sent to the labs of the Institute of History, Archaeology and Ethnology, Far-Eastern Branch, IHAE-FEB RAS Vladivostok, Russia (2017) and UMR 8096 Archéologie des Amériques in Paris (2018) for the further extraction of the starch (procedure detailed in the main text and in Longo et al. 2021, 2022).^5,6^ To perform morphometric evaluation, the stones were 3D scanned using the Artec3D Space Spider light scanner, while molds were obtained from both putative used and unused surfaces for comparative use-wear traces analysis^5,6,10^. Detailed functional analysis (G.S. PhD thesis) supports the intentional use of the GSTs in multiple sequential and contemporary utilization to process different materials, which includes ochre and starch-rich plant organs: USO and ASO^5,6,10^ (example of traces observed under 3D digital microscope Hirox KH-8700 accessed at the Cyprus Institute (2016-2017) are presented in Figure 2). Moreover, the areas with the carbonate crust still covering zones of some GSTs were also sampled for starch grains by carefully removing said crust using each time a clean scalpel, and the area underneath was then sonicated or soaked in carbonated water to dislodge the putative remains. The area was finally molded for surface texture analysis. Finally, control samples were taken by pipetting small amounts of deionized water from the inner surface of the wooden boxes containing the stone tools. As previously detailed in Longo et al. 2022,^6^ these wooden boxes were themselves encased in sealed metal boxes located on museum shelves. From the control samples, no starch grains were retrieved.

**Supplementary Fig. 2**. Brînzeni I, both a grinding stone (A) and a pestle (B) were refitted during the present study, intended as a good indicator that the stones remained untouched after their abandonment; in a second instance, the present refitting suggests that no attention by previous studies – devoted to the flaked industry – was paid to the macrolithic tool assemblage, leaving the pebbles almost untouched for further studies. Use-wear traces observed on the active surfaces of the GSTs as observed with Hirox 8700, using mid-range zoom lens with large depth-of-field and long working distance. A selection of different features affecting the stone’s working surface: polish (B-C, E, H) and striations (D-G, I-J).

2.2 **Surein I**

On the eastern slope of the Bel’bek gorge (Crimea Peninsula, near Sebastopol), the large rock shelter of Surein I (43 x 15 x 10 m) overlooks the second ridge of the Crimean Mountains at 110 m asl. Among the best-known Palaeolithic sites of Crimea, Surein I was first tested by K.S. Merezhkovski (alias Merejkowski) in 1879-1880 and then excavated between 1926-1929 by G. Bonch-Osmolovski, a renowned archaeologist who, in the same years, discovered the site of Kiik-Koba, the first site yielding Neanderthal remains in Crimea. Bonch-Osmolovski was also a highly skilled museum curator at the Department of Archaeology of the Peter the Great Museum of Anthropology and Ethnography (Kunstkamera) of the Russian Academy of Sciences, St. Petersburg, Russia (hereinafter MAE-RAS), where the lithic assemblage has been curated since then ^11,12^.

Pollen analysis performed at different Crimean sites, reveals a low-mountain forest–steppe environment. The western area of the Crimean Mountain is characterized by plateau-like tops, covered by meadow steppe with a prevalence of Poaceae and mesophytic herbs. The vegetation on the lower parts of the slopes has parallels with Brînzeni’s environment, otherwise for Surein I it is worth noting the occurrence of Fagaceae, well represented by several species of oak (Quercus pubescens, Q. petrae and Q. robur). In our reference collection both starches and fibers from other reported taxa in Crimean EUP sites are also present: hornbeam (Carpinus orientalis), maple (Acer campestre), hazel (Corylus avellana), spindle-tree (Euonymus europea), dogwood (Cornus mas and C. sanguinea), hawthorn (Crataegus pentagyna), and buckthorn (Rhamnus cathartica). ^2,11^

***2.2.1 Surein I Object’s biography****.* In order to reconstruct the object biography of the unique grinding stone retrieved at Surein I (See Supplementary Fig. 1), it has to be stressed the outstanding modernity of Bonch-Osmolovski' methodological approach to excavation, encompassing cutting-edge techniques that he applied at both the sites of Surein I and Kiik-Koba, the latter yielding the first human remains of Neanderthals in Crimea presented to the world in 1925 with an article in Science News.^13–17^

Thanks to the vanguard excavation and conservation techniques pioneered at Surein I, the context including the grinding stone, associated with flint artifacts and faunal remains (horse bones, still in anatomical connection^11,12^, was transported intact to the museum where it has ever since been protected under a glass case. However, after more than 100 years it is impossible not to consider putative management operations that occurred to the grinding stone tool since when it entered the museum collections, although its uniqueness was recognized since its early emergence and thereafter, and it is widely known until present days^18–20^. Although it is not possible to control the several steps of the curation process that occurred across the nearly 100 years, the organized composition of contextual presence of artifacts (flint flakes and large GST) and animal bones, make it the oldest and best-preserved evidence of the intentional structuring of the living space by early *Homo sapiens*. Its conservation conditions, always protected by a glass case, made it an excellent candidate for investigating its active surface. The large grinding stone was accessed by the authors at the MAE-RAS storage facilities during the 2015 and 2016 sampling campaigns, under an overall research agreement between NTU, Singapore and MAE-RAS.

The grinding stone – a large pebble composed of biogenic limestone, 236 x 122 x 68 mm – belongs to layer 3, the lowermost level indisputably attributed to the Aurignacian^18–21^, that also yielded a molar tooth attributed to *Homo sapiens*^11^, at present unavailable in the collections). New AMS radiocarbon dating on horse bone collagen - Beta 462734 carried out by the authors (2017, LL, unpublished data) is highly consistent with the reported oldest chronology.

The stone was analyzed in the storage room of the MAE RAS, St. Petersburg. Preliminary observation with Olympus BHMJ (metallographic microscope)^5,6^ were at the base of the identification of the working surface, which bears evident flattened and smoothed areas. Several molds were collected from this surface and used for both use-wear traces and associated residue analysis. Afterwards, the stone was immersed in bi-distilled water placed into an ultrasonic bath, and the obtained solution was stored in 50 ml tubes after adding few drops of ethanol. The solution was then processed and analyzed in the Vladivostok lab (I.P.).^5,6^ The molds where sonicated separately in a second time and processed in Paris since 2018.^6^ Traceological analysis was performed directly on the GST (N.S. and V.T.) and on molds (L.L. and G.S.), using the 3D digital microscope Hirox KH-8700 available at the Cyprus Institute (Nicosia, Cyprus) and a SEM. The analysis highlighted the presence of large flattened areas, smoothed and polished asperities and several groups of long striations, all features that are visible in Supplementary Figure 3 and detailed in ^12^.

**Supplementary Fig. 3**. Surein I, biogenic limestone grinding stone (A), part of a structure recognized during the 1926-27 excavation. The GST was found associated with horse mandibles, teeth and bones still in anatomical connection. The prompt recognition allowed the great care devoted to this exceptional find, creating ideal conditions for further studies. Use-wear traces observed on molds peeled-off from the active surfaces of the GSTs as observed with Hirox 8700, using mid-range zoom lens with large depth-of-field and long working distance. A selection of different features affecting the stones working surface: polish (B-D), E: residue (possibly a fiber on a polished area). Reference samples were taken from the inside of the glass-case covering the stone tool and from the original sediment still adhering to the horse bones sampled for the radiocarbon dating. These samples were processed according to the conventional workflow in two different labs (Vladivostok and Paris) and did not reveal exogenous starch grains^5,6,10,12,21^.

2.3 **Kostenki 14-Markina Gora**

The Kostenki 14-Markina Gora (K14) open air site represents the oldest evidence of the presence of *Homo sapiens* along the Don River, starting at least 36,000 years ago^22^. K14 is part of the rich Kostenki-Borschevo suite testifying to the intense inhabitation of the area for about 15,000 years^23–25^. Being one of the most intensively investigated sites of the EUP in the Western Eurasia, the relevance of its involvement in our study is twofold: on the one hand, the analysis of the SU-RBRs extracted from a selection of 9 ground stones – among a large assemblage of non-flaked industry recovered over the last fifty years – from Layers III and IV represents one of the oldest pieces of evidence of starchy plant processing enacted by *H. sapiens*. On the other hand, our data support that by applying an appropriate sampling and a sound analytical approach, even old museum collections – *legacy objects* ^9^ – can be worthwhile to investigate for veritable ancient starch candidates^6^.

Among the 9 GSTs under investigation, here we are presenting the pivotal results obtained from tool K14 #35 retrieved in the upper part of layer III, which dates between 30.1- 31.8 ka. This layer leans immediately above the Campanian Ignimbrite layer, which is used to date the oldest presence of *H. sapiens* at K14 around 36 ka (Supplementary Fig.1). Pollen analysis reveals the presence of a grass-dominated (Poaceae) landscape with *Artemisia* and Chenopodiaceae accompanied by Polygonaceae, Cyperaceae, and Chicoriaceae. Birch and yernik replaced spruce, while few pollen also of *Alnaster* and *Salix* were found in ^24^.

The surface of K14 #35 was preliminary observed with metallographic microscope at IHMC, RAS in St. Petersburg. The identified used areas were molded for wear traces analysis, performed with the 3D digital microscope (Hirox KH-8700) at the Cyprus Institute (Nicosia, Cyprus), and SEM at NTU Singapore. The analysis revealed the presence of flattened areas, polishing of the crystal surfaces, battering marks, and fractures. Crystals present sharp edges symptomatic of contact with a rigid medium (Supplementary Fig. 4) Moreover, during the scanning of the molds, starches (ASC-1) still adhering to the surface were noticed. In order to extract the granules, the peels were sonicated and the extracted pellets analyzed with the SEM (ASC-3).

One soil sample associated with the relevant archaeological context was analyzed. Three starch grains were recovered from this particular control sample. Moreover, two samples of the volcanic ashes attributed to CY5 were analyzed, revealing the absence of starches confirming the absence of pollens already observed by Velichko et al.^24^

***Kostenki 14-Markina Gora Object’s biography****.* The SR-FTIR analysis was carried out on a small grinding stone – K14 Layer III L 33 214-225 #35 – made of fine-grained quartzite, a raw material available in the ravines nearby the site. Interestingly for our research is the fact that since 1984, Melekestsev and colleagues^26^ recognized the ash layers as the result of the catastrophic eruption of the Campi Flegrei (CY5) and therefore the oldest sediments of Kostenki 14 yielding the burial were split in layers III and IVb^22,23^. Our sampling focused on a selection of 9 ground stones from both the layers. A selection of the Upper Paleolithic ground stone collections, widely known among the Russian prehistoric community since almost all the stones are already described or mentioned in the Russian literature, is reviewed in a recent paper in English^27^. Therefore, it must be said that the handling and management of the Kostenki-Borshevo stone tools can be considered quite random and promiscuous and we acknowledged that biogenic residues we observed should be considered critically.

Kostenki 14 is considered a temperate steppe site where horse, mammoth and bison herds roamed and were hunted by early groups of modern humans around 36 ka ago. The pebbles retrieved at the site underwent xerophytic steppe conditions since their burial^28,29^. This environment is named *yedoma*, a cold, organic-rich permafrost dating to the Pleistocene. The longstanding cold to freezing conditions during the excavation, along with their subsequent curation in museum dark store-rooms – of the State Archaeological Museum-Reserve in Voronezh^25,30^. These situations (low temperatures and dark rooms) created the condition to “buy time”^9^ in favor of the conservation of biogenic residues. The cobbles from the Kostenki-Borshevo suite are usually deposited in the storage infrastructure designed by Rogachev since the early seventies^25^, although we accessed the ground stone tools at the IHMC-RAS in St. Petersburg (2015 and 2016) where the GSTs were loaded in wooden boxes and bagged separately, hence preserved from dust.

**Supplementary Fig. 4**. Kostenki 14, L 33 214-225 #35 retrieved in Layer III. B: aligned polish, C: tip of the crystals damaged by rounding and polishing, D: the same area as seen in 3D showing the microtexture roughness; E: 3D well developed polish. All the images were taken with an Hirox 8700, using mid-range zoom lens with large depth-of-field and long working distance.

3. **Sample Preparation**

**Supplementary Fig. 5.** A graphic representation of the U-RBR extraction pipeline.

4. **Spectra subtraction**

**Supplementary Fig. 6.** An example of the procedure for the subtraction of the contribution of the material surrounding ASC-2 particles is shown in the present figure. The average spectrum of the material surrounding the identified ASC-2 (red line) was subtracted from the raw spectrum of ASC-2 (blue line) applying a multiplicative coefficient estimated for minimizing the soil carbonate band, peaked at ~1410 cm^-1^. The resulting spectrum, representing the closest approximation to clean ASC spectrum, is shown by the black line.

**References and Notes**

1.	Demidenko, Y. Demidenko 2014 - The Great North Black Sea region Early UP and human migrations into the region from different territories IN ERAUL 140 + ArcheoLogiques 5, Liege.

2.	Gerasimenko, N. Environmental changes in the Crimean mountains during the Last Interglacial–middle pleniglacial as recorded by pollen and lithopedology. *Quaternary International* **164–165**, 207–220 (2007).

3.	Hardy, B. L. Climatic variability and plant food distribution in Pleistocene Europe: Implications for Neanderthal diet and subsistence. *Quaternary Science Reviews* **29**, 662–679 (2010).

4.	*Periglacial Landscapes of Europe*. (Springer International Publishing, 2022). doi:10.1007/978-3-031-14895-8.

5.	Longo, L. *et al.* A Multi-Dimensional Approach to Investigate Use-Related Biogenic Residues on Palaeolithic Ground Stone Tools. *Environmental Archaeology* (2021) doi:10.1080/14614103.2021.1975252.

6.	Longo, L. *et al.* Coupling the beams: How controlled extraction methods and FTIR-spectroscopy, OM and SEM reveal the grinding of starchy plants in the Pontic steppe 36,000 years ago. *Journal of Archaeological Science: Reports* **41**, (2022).

7.	Allsworth-Jones, P., Borziac, I. A., Chetraru, N. A., French, C. a. I. & Medyanik, S. I. Brînzeni: A Multidisciplinary Study of an Upper Palaeolithic site in Moldova. *Proceedings of the Prehistoric Society* **84**, 41–76 (2018).

8.	Sorrentino, G. *et al.* Tracing Old Gestures: A Multiscale Analysis of Ground Stone Tools Developed on Sequential Lab-Controlled Replicative Experiments. *Heritage* **6**, 4737–4767 (2023).

9.	Koh, A. J. & Birney, K. J. Ancient Organic Residues as Cultural and Environmental Proxies: The Value of Legacy Objects. *Sustainability* **11**, 656 (2019).

10.	Natalia Skakun *et al.* Grinding Stones and Pestles from the Szeletian Layer of Brînzeni-1 Grotto (Republic of Moldova) (in the Light of Experimental and Traceological Study, Data of Organic Remains Analysis and 3D Scanning). *Eminak* **0**, (2020).

11.	Vekilova, E. A. Surein I and its place among the Paleolithic sites of Crimea and the next territories. *Materials and Research in the Archaeology of the USSR (Materialy i Issledovaniya po Arkheologii SSSR)* **59**, 235–323 (1957).

12.	Longo, L., Skakun, N. N., Pantyukhina, I. E., Terekhina, V. V. & Sorrentino, G. Aurignacian grinding stone from Surein I (Crimea): “trace-ing” the roots of starch-based diet. *Journal of Archaeological Science: Reports* **38**, 102999 (2021).

13.	Crimean Neanderthal Remains. *Science* **61**, (1925).

14.	Bonch-Osmolovsky, G.A. Paleoliticheskaya stoyanka v Krymu. Predvaritel’noye soobshcheniye [Paleolithic site in the Crimea. Preliminary Report]. *Rossiyskiy antropologicheskiy zhurnal [Russian Journal of Anthropology]* 81–87 (1926).

15.	Bonch-Osmolovsky, G.A. Le paléolithique de Crimée. Akademia NAUK SSSR, Leningrad. *Separate impression of Bulletin of the Commission for the study of the Quaternary period 1* (1929).

16.	Bonch-Osmolovsky, G.A. The study of the Crimean caves in 1932. *PIMC* **9–10**, 56–57 (1932) (in Russian).

17.	Bonch-Osmolovsky, G.A. Itogi izucheniya krimskogo paleolita [The results of studies of the Crimean Palaeolithic]. In: Trudi II Mezhdunarodnoi konferenzii, Sovetskakya sekziya po isucheniu chetvertichnogo perioda v Evrope 5 [Proceedings of the international conference of the Association for the study of the Quaternary period in Europe. Soviet section] 5, 124–130 (in Russian). *Trudi II Mezhdunarodnoi konferenzii, Sovetskakya sekziya po isucheniu chetvertichnogo perioda v Evrope 5 [Proceedings of the international conference of the Association for the study of the Quaternary period in Europe. Soviet section]* 124–130 (1934).

18.	Bataille, G., Tafelmaier, Y. & Weniger, G. C. Living on the edge – A comparative approach for studying the beginning of the Aurignacian. *Quaternary International* **474**, 3–29 (2018).

19.	Bataille, G. Extracting the ‘Proto’ from the Aurignacian. Distinct Production Sequences of Blades and Bladelets in the Lower Aurignacian Phase of Siuren I, Units H and G (Crimea). *Mitteilungen der Gesellschaft für Urgeschichte* **25**, 49–86 (2016).

20.	Zwyns, N. Small laminar blanks at Siuren I Rockshelter: Technological and comparative approach. in 359–373 (2012).

21.	Skakun, N. *et al.* Results of the integrated study of a large stone tool from the Upper Paleolithic site Suren I. *Camera Praehistorica* 8–24 (2021) doi:10.31250/2658-3828-2021-1-8-24.

22.	Dinnis, R. *et al.* New data for the Early Upper Paleolithic of Kostenki (Russia). *Journal of Human Evolution* **127**, 21–40 (2019).

23.	Sinitsyn, A. A. & Hoffecker, J. F. Radiocarbon dating and chronology of the Early Upper Paleolithic at Kostenki. *Quaternary International* **152–153**, 164–174 (2006).

24.	Velichko, A. A., Pisareva, V. V., Sedov, S. N., Sinitsyn, A. A. & Timireva, S. N. Paleogeography of Kostenki-14 (Marinka Gora). *Archaeology, Ethnology and Anthropology of Eurasia* **37**, 35–50 (2009).

25.	Rogachev, A.N. *On the complex gathering as a form of economy in Palaeolithic epoch on the Russian plain*. (Lebedinskaya, G.V., Rabinovich, M.G. (Eds.), 1973).

26.	Melekestsev, I. V, Kir’ianov, N. D & Praslov, N.D. Katastroficheskoe izverzhenie v rajone Flegrejskukh polej (Italiya). *Vulkanologiya i sejsmologiya* 34–35 (1984).

27.	Stepanova, K. Upper Palaeolithic grinding stones from Eastern European sites: An overview. *Quaternary International* **541**, 162–181 (2020).

28.	Rogachev, A.N & Sinitsyn, A.A. Kostenki 14. in *Paleolit Kostenkovsko-Borshchevskogo raiona na Donu 1879–1979* 145–162 (1982).

29.	Spiridonova E.A. Palinologicheskoye obosnovaniye vozrasta otlozhenii stratigraficheskoi kolonki stoyanki Kostenki-14 (Markina gora). *In Osobennosti razvitiya verkhnego paleolita Vostochnoi Evropy. St. Petersburg.: Izd. IIMK* 237–246 (2002).

30.	The State Archeological Museum-Reserve Kostenki. http://www.russianmuseums.info/M655.
